# Ethical leadership and workplace behavior in the education sector: The implications of employees' ethical work behavior

**DOI:** 10.3389/fpsyg.2022.1040000

**Published:** 2023-01-04

**Authors:** Fengrui Guo, Zhongyi Xue, Jiaxu He, Fakhra Yasmin

**Affiliations:** ^1^School of Marxism, Dalian Maritime University, Dalian, Liaoning, China; ^2^School of Education, South China Normal University, Guangzhou, China; ^3^Department of Informatics and Quantitative Methods, University of Hradec Kralove, Hradec Králové, Czechia

**Keywords:** organizational commitment, ethical work behavior, education sector, ethical leadership, China

## Abstract

The study aimed to examine the effect of ethical leadership on employees' ethical work behavior. Furthermore, this study examined the mediating role of organizational commitment in the relationship between ethical leadership and employees' ethical work behavior. This study was conducted in a public-sector educational organization, and 500 questionnaires were distributed among targeted employees. Out of these, 400 valid responses were received from individuals working in the education sector in China. The findings showed a positive and significant impact of ethical leadership on employees' ethical work behavior. We found that organizational commitment also significantly mediates the relationship between ethical leadership and employees' ethical work behavior. The practical implications of the current study are useful for all organizations in the public sector. As ethical leadership is positively related to employees' work behavior, we recommend that organizations should develop and conduct such training programs to promote ethical work behavior. Leaders with a strong sense of ethics should be hired to encourage ethical work behavior within the organization. Furthermore, organizations can conduct management training programs, seminars, and workshops to encourage such behavior. Ethical behavior can be encouraged among employees by making it a clear requirement of their jobs. To achieve positive results, top management and leadership must educate employees on the value and importance of ethical behavior in the workplace.

## 1. Introduction

Recent ethical scandals have drawn attention to the importance of and need for further research on the ethical behavior of leaders and followers, especially in the public sector. The results presented by the 2018 federal employee viewpoint survey in the USA show that the value of honesty and integrity was not maintained by their leaders, as 45% of government employees and 34% of private-sector employees were reluctant to expose violations of rules and regulations due to fear of retaliation. Brown and Treviño (2006) defined ethical leadership (EL) as the “demonstration of normatively appropriate conduct through personal actions and interpersonal relationships and their promotion of such conduct to followers through two-way communication, reinforcement, and decision making.” EL is demonstrated to be a leadership style that helps leaders treat their subordinates fairly, honestly, and respectfully. This behavior encourages them to be ethical and take more initiatives, which ultimately helps accomplish organizational goals. Brown and Treviño (2006) claimed that ethical leaders are eager to enhance the ethical behavior of their followers by providing guidance, communicating moral standards, and having a sense of responsibility regarding their conduct. Over the past decade, more attention has been given to demonstrating and studying the meaning of EL and its results by adopting systematic approaches (Hassan et al., 2014; Fehr et al., 2015).

Organizations focus on research and development for business growth to gain a competitive advantage over their rival organizations. This concept strongly emphasizes the synchronization of the organization–human relationship to achieve maximum productivity (Huo et al., 2022). The relationship between leaders and subordinates is meant to be stronger if leaders maintain respect, care, and honesty regarding decisions related to employees' wellbeing (Li et al., 2022). Such moral behavior of supervisors serves as a driving force for followers, which enhances organizational commitment (OC) and positively improves employees' ethical work behavior (EWB). The ethical aspects of leadership have received greater consideration from academia and researchers based on the recurring ethical scandals both in public- and private-sector organizations in recent years (Ahmad et al., 2021; Banks et al., 2021; Koay and Lim, 2021; Qasim et al., 2021). Martin et al. (2022) investigated the role of EL in team efficacy. Eluwole et al. (2022) stated that EL leads to employee outcomes.

Ethical leaders are reliable role models for their followers and treat them with respect and dignity. Many scholars describe them as being honest, caring, unbiased decision-makers who are also involved in ethical aspects that encourage subordinates' EWB by providing them with rewards, recognition, and penalties depending on their behavior (Qing et al., 2020; Zeb et al., 2021; Idrees et al., 2022). In the present study, we focused on the mediating role of OC in the EL-EWB link. Some researchers recently studied the mediating role of OC (Akhtar et al., 2019; Ashfaq et al., 2021; Choudhary and Saini, 2021; Donkor et al., 2021).

To avoid economic crises and repeated ethical scandals, the field of business work ethics has undergone numerous transformations to promote transparency and ethical behavior improvement. Formal and informal ethical codes of conduct were developed and implemented to enhance EWB in the workplace, but the ethics code alone cannot ensure EWB. As we observed in Islamic culture, especially in China's public-sector organizations, there is an increasing trend in ethical scandals, and many unethical conducts of employees have been reported. These behavioral issues are significantly contributing to the downfall and financial loss of government organizations (Yasir and Rasli, 2018; Aslam et al., 2021). The current economic condition of China is at its worst, and almost every public sector organization is facing a crisis and, therefore, is not generating profits to support the country's economy. The unethical behavior of employees in the workplace is rapidly increasing in China, causing financial loss and other organizational-level issues, especially in public-sector organizations. This includes leaving the office early (66%), taking longer breaks for lunch (90%), misusing official computers for gaming, entertainment, and social media instead of work (49%), stealing office equipment (49%), and making unofficial calls from office telephones (94%) (Bashir et al., 2012). The behavior of ethical leaders and their traits can transform their employees' perceptions of their work, their beliefs, and their views about the context of work. This transformation in their behavior will motivate them to exert extra effort in their work and avoid unethical conduct at the workplace.

It is essential to explore the factors that significantly affect the emergence of ethical behaviors in the workplace for the achievement of organizational objectives in order to overcome the economic crisis. Due to the severity and intensity of ethical behavior in the workplace, this research will contribute to the understanding and implementation of work ethics to enhance the ethical behavior of leaders and employees in China's public sector organizations.

The current study observed the causal-effect relationship between the variables, which is exploratory. This study contributes to the literature on behavioral research by further studying different constructs that contribute to shaping employees' behavior in a different field. It will serve as a guide for measuring the effect of EL on EWB by using a different mediator and moderator variables. This study is also helpful in different settings: the study findings, from an educational point of view, are expected to help higher educational institutions sustain their moral values and norms to foster an ethically rich culture. It will also help them to understand their role in national development and how they provide relevant knowledge to their students. Educational institutes should provide knowledge related to ethical and moral values, which we have also learned from our religious teachings. Currently, many higher education institutes are focusing solely on the results their students achieve. Nevertheless, they should motivate their faculty and students to practice an ethical code of conduct, which, ultimately, also helps shape the ethical behavior of society at large.

## 2. Literature review

### 2.1. Social learning theory

Social learning theory stipulates that people in a given society aim to learn from and imitate the behavior of those with superior social status and higher ranks (Bandura, 1969; Bandura and Walters, 1977). The behavior of employees toward their leader and coworkers reflects the same behavior because they imitate and learn it. Being better at taking accountability for their actions, ethical leaders make it clear to their teams what the organization's goals are and what is expected of them (Kalshoven and Den Hartog, 2009). First, employees also behave similarly by imitating, learning, and helping others achieve the organization's ultimate goals. EL consists of two essential and associated dimensions in which the leader first sets the ethical standards and then provides rewards, recognition, and penalties to ensure these standards are followed. Second, these leaders exhibit desirable qualities such as honesty, integrity, credibility, care, and respect for other employees through their behavior in the workplace (Yang and Wei, 2017; Huo et al., 2022). A leader observes the behaviors of other people and the outcomes of such behaviors to learn ethical standards, acquires related knowledge by evaluating the information, and retains the processed information. Furthermore, a formal code of conduct can be developed as a standardized guideline for developing ethical behavior in the workplace.

### 2.2. Ethical leadership and EWB

Ethical leaders develop a positive and high-quality relationship with their followers by respecting, caring for, and valuing their followers' beliefs and making decisions with their followers' wellbeing in mind; this encourages their followers to behave ethically in the workplace (Zhu et al., 2004). Many previous studies indicated that ethical leaders encourage constructive emotions and a sense of belongingness among their followers, which results in them developing a strong bond with the leader's beliefs, values, and targets (Ribeiro et al., 2018). Pastin (1988) also demonstrated in his study that corporations have given attention to EL because it will foster a culture of weak organizations and establish a good relationship with their employees. When ethical leaders treat employees through two-way communication to show them that they are part of the organization, employees become motivated to engage and achieve the organization's overall objectives (Babalola et al., 2019). Cognition plays a vital role in learning when employees observe the behaviors of their leaders. Social learning theory demonstrates that employees learn through observations, imitations, and modeling; employees learn by observing attitudes, behaviors, and the results of others' behaviors. Thus, the employees gradually behave accordingly by imitating the behavior of their role models.

The study by Kalshoven et al. (2016) indicated that ethical leaders work efficiently because their expectations toward employees are clear. Furthermore, they indicated that an ethical leader communicates openly and fairly, influencing a positive relationship with the employees' behavior. Okan and Akyüz (2015) suggested that EL predicts some outcomes, such as the leader's effectiveness, job satisfaction, devotion to exert more effort, and readiness to report any ethical problems. Understanding its mutual benefits and involving employees in the decision-making process to achieve organizational objectives shape the EWB of employees. It promotes positive work behavior, as they feel that their leader is trustworthy and fairly involved in helping them achieve the targets (Brown and Treviño, 2006).

Meyer et al. (1993) explained that ethical leaders serve as role models, encouraging integrity, dignity, credibility, and trust within the organization; the followers reciprocate the ethical behavior with high OC. When an ethical leader discusses moral values and business ethics with their followers and acts as a model for them by demonstrating how to perform in the right way, the followers reciprocate the same behavior by imitating the traits of their leaders. Leaders can precisely identify which behaviors encourage EWB. Then, they will attempt to modify their behaviors to treat their subordinates fairly and without bias in order to make them feel good (Brown and Treviño, 2006).

Moreover, Yang and Wei (2017) indicated that EL positively influences employee behavior toward work. They further added that EL establishes EWB by listening to and keeping the best interests of subordinates in mind while making a decision. The followers understand that strict disciplinary action will be taken against them if they are involved in unethical conduct and violate the organization's ethical standards. A leader's positive gesture enhances employees' motivation to do their work more efficiently and effectively. Furthermore, many previous studies indicated that EL is positively influenced by various dimensions of leadership effectiveness toward employees' enhancement, commitment, and work performance as well (Yang and Wei, 2017; Babalola et al., 2019; Qing et al., 2020; Banks et al., 2021; Koay and Lim, 2021).

Belschak et al. (2018) asserted that there are various suitable ways to influence EWB among employees. They further added that, in this way, employees feel more confident about their job requirements and efficiently perform them. Some other researchers suggested that employees who have a positive relationship with ethical leaders are more likely to develop an inspirational goal and even lead their organization to tremendous success (Akhtar et al., 2020a, 2022a; Javed et al., 2021). Furthermore, some previous studies also supported the idea that ethical leadership helps employees attain organizational objectives more confidently as they are committed, sincere, and honestly follow the instructions given by their leaders (Ahmad et al., 2021; Banks et al., 2021; Koay and Lim, 2021).

H1: Ethical leadership is positively related to EWB.

### 2.3. Mediating role of OC

The OC influences as a mediator and has the power to enhance EWB in the organization (Mowday, 1979). Akhtar et al. (2022b) recommended that some diverse employees have dissimilar norms, values, customs, and cultures, and it is the organization's responsibility to make them feel safe and positive. They further added that, in this way, employees feel that employees become an essential member of their organization, thereby making them more committed to it.

Brown and Treviño (2006) elucidated that the attributes of ethical leaders make them more valuable and attractive among their followers. Furthermore, the followers receive more respect, consideration, and support from their leaders, making them feel highly grateful and thus, developing a positive attitude with greater job satisfaction and commitment. Indeed, ethical leaders treat employees fairly and equally while making important decisions regarding job design and related activities. These attributes provoke enthusiasm and trust among followers, which contribute to OC (Ko et al., 2020). Lapointe and Vandenberghe (2018) also concluded in their study that OC was also significantly influenced by organizational and personal factors, which are interconnected with emotions and emotional reactions and, as a result, affect organizational productivity and productivity performance.

Qing et al. (2020) elucidated in their study, which was conducted in the public sector of China, that employee attitudes are positively influenced by EL, making their commitment and loyalty toward their organization stronger and more robust. They further added that EL positively impacts an organization and its employees' work behaviors. Observational learning theory suggests that when ethical leaders give rewards and punishments, it increases and decreases the chances of behaviors recurring. Brown and Treviño (2006) discovered that when organizations maintain their moral values and principles by implementing EL, employees' OC and loyalty are positively enhanced. They further added that EL has a positive and significant impact on OC, and the turnover ratio of employees is significantly reduced. Such committed employees ultimately strengthen the ethical culture of organizations and positively impact employees' work behavior. Similarly, unethical behavior of leaders weakens organizational culture and subordinates' commitment, which negatively impacts work behavior.

H2: OC mediates the relationship between EL and EWB.

## 3. Research methodology

This section explains the methodology of this study, which includes the nature of the study, research design and approach, method of data collection, and questionnaire design. Furthermore, this section describes the target population, unit of analysis, sampling technique, and sample size. It also explains the techniques of data analysis that have been used in this thesis. The study's research model was developed within the framework of theoretical assumptions and antecedent literature, which suggest that EL would be effective on followers' EWB and OC. In contrast, OC plays an intermediary role in the relationship between EL and EWB, making the current study's design essential to determining cause and effect.

We used a deductive approach to test the implications of social learning theory on EL and EWB. The positivist social and quantitative approaches have been used in this study. The current study was based on a cross-sectional time horizon and mono method. The survey technique was applied to measure the impact of all constructs. The present study investigated the impact of EL on EWB in public-sector organizations in China. Therefore, it is also pertinent to determine the unit of analysis before deciding on the target population. Previous studies used it (Akhtar et al., 2020b, 2022c; Syed et al., 2021; Huo et al., 2022).

The target population is public-sector organizations, where the public directly interacts with employees on a daily basis, and employees frequently interact with their managers and reporting officers. Therefore, EWB matters greatly in such organizations for better customer service and performance. We selected four universities that have more than 10,000 employees. The purposive or judgmental sampling technique was cost-efficient, time-effective, and helpful in approaching the target quickly. In the current study, only public-sector organizations were selected for data collection, and the sample size was determined as around 400 respondents with a 95% confidence level and a *p* = 0.5 from the overall population of four organizations (Yamane, 1973).

### 3.1. Variable measurements

#### 3.3.1. Ethical leadership

This study used a 10-item scale to measure the ethical behavior of leaders developed by Brown and Treviño (2006). The scale is reliable because Cronbach's alpha is 0.814. We used a five-point Likert scale to measure the items. Sample items included “Discusses business ethics or values with employees.”

#### 3.3.2. OC

The 18-item scale OC developed by Allen and Meyer (1993) was used. The scale is reliable because Cronbach's alpha is 0.747. We used a five-point Likert scale to measure the items. Sample items were, “I would be very happy to spend the rest of my career with this organization.”

#### 3.3.3. Ethical work behavior

To measure ethical behavior in the workplace, a 13-item scale was used, which was developed by Loe et al. (2000). This scale is reliable as Cronbach's alpha is 0.745. We used a five-point Likert scale to measure the items. Sample items included “I think my coworkers do not pass the blame for errors on to an innocent colleague.”

## 4. Data analysis

The current study distributed questionnaires to 500 employees of public-sector organizations, of which 430 responses were collected. These collected questionnaires were thoroughly checked; the excluded questionnaires had missing values. A total of 400 valid responses were received, with an 80% response rate.

The findings reveal that the percentage of men who participated in this survey was 82%, while only 18% of women participated. The employees' participation level regarding age group in the survey represents that 27.8% were in the 21–30 age group, 59.8% were in the 31–40 age group, 11% were in the 41–50 age group, and 1.5% belonged to the 51 and above age group. Regarding the education level, 6.3% were intermediate employees, 53% were graduate employees, 40.3% were postgraduate employees, and 0.5% had a doctorate. A total of 74.8% of government employees participated in this survey, and 25.2% were semi-government department employees. We used gender, age, and education as control variables, as recent studies have used them (Syed et al., 2020; Li et al., 2022).

The findings in Table 1 reveal a positive correlation between EL and EWB (*r* = 0.627, *p* = 0.000), EL and OC (*r* = 0.639, *p* = 0.000), and OC and EWB (*r* = 0.697, *p* = 0.000).

**Table 1:** Correlation analysis.

	**Mean**	**SDs**	**EL**	**EWB**	**OC**
**EL**	**4.09**	**0.46**	**(0.814)**		
EWB	4.04	0.37	0.627^**^	(0.745)	
OC	4.08	0.31	0.639^**^	0.697^**^	(0.745)

^**^Correlation is significant at the 0.01 level (2-tailed).

### 4.1. Hypotheses testing

The results showed that EL has a positive influence on EWB (*B* = 0.248, *t* = 6.95, *p* = 0.04). As a result of the preceding findings, Hypothesis 1 is verified. As per H2, OC plays a mediating role between EL and EWB. Thus, according to Table 2, OC mediates the effect of EL on EWB (*B* = 0.258, *SE* = 0.039, CI = 0.19, 0.34), as both confidence interval boundaries did not contain zero, supporting H2.

**Table 2:** Mediation results.

**Variable**	**R^2^**	**B**	**SE**	**T**	***p***
**EL effect on EWB**	**0.39**	**0.25**	**0.04**	**6.95**	**0.001**
EL effect on OC	0.41	0.43	0.03	16.56	0.001
OC effect on EWB	0.54	0.60	0.05	11.36	0.001
**Bootstrap results for indirect effects**				
		**Effect**	**SE**	**LL 95% CI**	**UL 95% CI**
OC		0.26	0.03	0.19	0.34

N = 400.

## 5. Discussion

This study aimed to explore the empirical and theoretical relationships between EL and EWB, where OC is used as a mediator for public-sector employees. Our findings also confirmed the conclusion of previous research where the effect of EL on OC and EWB was explored (Qing et al., 2020). The study findings confirmed the first hypothesis of the current study, i.e., EL is positively related to employee EWB, confirming that leaders who practice EL have a significantly positive impact on their EWB. The employees tend to reciprocate the ethical behavior when they recognize that they are treated equally and fairly by their supervisors and leaders. Ethical leaders influence employees' behavior by making fair and balanced decisions and involving them in the decision-making process. Hence, the study findings supported the arguments and confirmed the findings of previous studies by demonstrating that, when leaders keep the best interests of subordinates in mind and properly listen to what they want to say, it will ultimately develop trust and altruism and promote EWB. These leaders are trustworthy and honest and encourage ethical behavior among their subordinates by communicating moral standards and punishing employees who violate these ethical standards (Yang and Wei, 2017). Leaders are able to precisely identify which behaviors motivate EWB. They will modify their behaviors to treat their subordinates fairly and without bias to make them feel good (Brown and Treviño, 2006). Engelbrecht et al. (2017) also discovered that EL improves employee behavior.

The study findings for the second hypothesis of the current study, i.e., OC mediates the relationship between EL and employee EWB, confirm the previous research findings that OC positively mediates the association between EL and employees' work behavior. Meyer et al. (2002) explained that ethical leaders promote integrity, dignity, credibility, and trust within the organization by presenting themselves as role models, and the followers reciprocate the ethical behavior with a high OC level. Okan and Akyüz (2015) suggested that EL predicted some outcomes like a leader's effectiveness, OC, devotion to exert more effort, and readiness to report the ethical problem. Furthermore, they indicated that an ethical leader communicates openly and fairly, influencing positive behavior toward employees. Engelbrecht et al. (2017) also concluded that ethical leaders improve employee behavior. They further added that EL establishes an EWB work environment within organizations. Furthermore, many previous studies indicated that EL is positively influenced by various dimensions of leadership effectiveness toward employees' enhancement, commitment, and work behavior (Hassan et al., 2014; Yang and Wei, 2017; Ribeiro et al., 2018; Babalola et al., 2019; Qing et al., 2020; Banks et al., 2021; Donkor et al., 2021; Koay and Lim, 2021).

### 5.1. Practical implications

The following are the practical implications of the study. First, this study established that EL is valuable in developing OC and EWB. This study also suggested that the role of EL is essential to providing appropriate guidance in ethical dilemmas to encourage EWB. Second, the study's findings revealed that EL has a strong, positive impact on employees' work behavior. Thus, it is anticipated that leaders and organizations should ascertain such situations in which they can develop employees' work behaviors. It is further suggested that organizations should conduct seminars and conduct training sessions to boost employee EWB. Third, as the EL has a significant and positive impact on OC and EWB, organizations must encourage ethical behaviors among their employees. For example, organizations can develop and hire leaders with a strong, wise vision of moral obligations.

Furthermore, organizations can conduct management training programs and workshops to encourage such behavior. Fourth, ethical behavior can be encouraged among employees by making it part of their job requirements. The organization can display on information boards that such behaviors will be adequately punished or rewarded so that all employees feel obligated to perform ethically. To achieve positive results, top management, and leadership must educate employees on the value and importance of ethical behavior in the workplace. Finally, the organizations can develop a proper ethical code of conduct and devise policies accordingly to enhance EWB among subordinates.

### 5.2. Limitations and future directions

The core objective of the research was to explore the impact of EL on EWB. First, we used only one mediator, which performed a significant mediating role between the EL and EWB. Second, data were collected only from China. Third, the only participants in this study were employees of public-sector organizations. Private firms could not be included in this study. Fourth, a self-reported bias may exist, as the respondents were supposed to answer questions about the ethical behavior of their coworkers and leaders. Since this study was conducted using the quantitative approach, future researchers should use the mixed-methods approach to further explore the relationship among the variables. Fifth, the scope of the research can be enhanced by examining other areas of Asia. Sixth, this research can also be conducted in China's business, corporate, and private sectors. Finally, the model can also be applied to other sectors, cultures, contexts, and countries.

### 5.3. Conclusion

This study extended the discussion on the impact of EL on the EWB of public-sector employees in the context of China. The study findings confirmed the acceptance of the stated hypothesis, which explains that EL enhances EWB. Furthermore, OC performs an important role as a mediator and promotes the relationship between EL and EWB. By emphasizing the significance of EL, the findings of this study can help employees enhance EWB. It also helps scholars and practitioners better understand the impact of OC on EWB from the viewpoint of developing countries. Further, the finding indicates that the EL and OC have a significantly positive impact on the EWB of employees in China. Much recent research, however, has been conducted to explore the impact of EL in developed countries. EL, OC, and their impact on EWB vary across nations. This study is helpful and provides a baseline for scholars and practitioners to study this phenomenon in different cultures and sectors, especially in growing and developing countries. Hence, this study will help them recognize the significance of EL, and its implementation in every sector will become necessary for EWB.

## Data availability statement

The raw data supporting the conclusions of this article will be made available by the authors, without undue reservation.

## Ethics statement

The studies involving human participants were reviewed and approved by Ethics Committee of Dalian Maritime University. The patients/participants provided their written informed consent to participate in this study.

## Author contributions

FG: investigation, writing, and conceptualization. ZX: conceptualization and methodology. FY: investigation, software, formal analysis, and writing—original draft. JH: investigation and writing—review and editing. All authors contributed to the article and approved the submitted version.
